# Community Health Literacy Assessment: A Systematic Framework to Assess Activities, Gaps, Assets, and Opportunities for Health Literacy Improvement

**DOI:** 10.3928/24748307-20190821-01

**Published:** 2019-10-10

**Authors:** Heather Platter, Katya Kaplow, Cynthia Baur

## Abstract

**Background:**

The National Action Plan to Improve Health Literacy makes the case that a wide range of organizations and professionals must work together to improve health information and services to achieve a health literate society. The context and framework for this collaboration and action, however, have yet to be well-articulated. We report on our use of a community health needs assessment model to describe county and state health literacy activities, gaps, assets, and opportunities. This approach combines the public health best practice of learning about communities through systematic assessments and the emerging health literacy best practice of studying organizational behaviors.

**Brief description of activity:**

A community health literacy assessment was implemented from January 2018 to April 2018. The purpose was to collect information about county and state-level health literacy activities, gaps, assets, and opportunities. We used this information to characterize the status of health literacy in Maryland and establish an initial baseline for county and state strategic planning work and future collaboration.

**Implementation:**

An environmental scan of each county in Maryland identified health indicators, community resources, and health organizations or professionals. Organizational representatives participated in interviews about their health literacy work. Interviews were analyzed to identify themes as well as summarize and quantify perspectives by county. We convened a forum, disseminated preliminary findings, and performed member checking to assess agreement with the results.

**Results:**

The team interviewed 56 individuals from 49 organizations. Themes of health literacy definitions as well as organizational ranking on the use of health literacy best practices are discussed in this article. Forty public health professionals, including 10 interview participants, attended the forum. Member checking assessed interview participants' agreement with results and interpretations, which were found to be accurate portrayals of their responses.

**Lessons learned:**

Lessons learned include being flexible with the interview approach, performing member checking, and allowing participants to self-define health literacy. Our experience shows a small team can perform a large-scale assessment that provides actionable information at state and county-levels. The results can influence future interventions, inform strategic planning and collaboration, and lead us toward a health literate society. **[*HLRP: Health Literacy Research and Practice*. 2019;3(4):e216–e226.]**

**Plain Language Summary:**

A systematic community health needs assessment framework was used to collect information about health literacy activities, assets, gaps, and opportunities at the state and county level. Participant feedback showed the team accurately captured the activities, assets, gaps, and opportunities to improve health literacy practices. A needs assessment framework is feasible for describing community health literacy.

The basic argument of the National Action Plan to Improve Health Literacy is that limited health literacy is a society-wide problem that requires coordinated, comprehensive, multilevel approaches and solutions to see measurable, lasting improvements (U.S. Department of Health and Human Services, 2010). The U.S. National Assessment of Adult Literacy (NAAL) results established that only 12% of adults have proficient health literacy skills, demonstrating the critical need to improve the nation's health literacy on a massive scale (Kutner, Greenberg, Jin, & Paulsen, 2006). The National Action Plan calls for policymakers, public and private sector organizations, community-based organizations, and health care and education professionals to work together and use best practices to improve the nation's health literacy and create a health literate society (U.S. Department of Health and Human Services, 2010).

Although new frameworks and tools exist for organizations to assess their own health literacy practices and policies, nothing comparable is available to guide multiple collaborating organizations or communities that want to make health literacy improvement a priority (Farmanova, Bonneville, & Bouchard, 2018). In addition, the field lacks a robust set of examples and best practices for community health literacy interventions (Baur, Martinez, Tchangalova, & Rubin, 2017). Yet, it is in neighborhoods, cities, counties, and states where many organizations, activities, and resources must come together under a health literacy banner to develop the health literate societies envisioned in the National Action Plan.

## Background

Population surveys have been one common method of characterizing health literacy in communities or groups defined, for example, by race/ethnicity or geography. Six states participated in the 2003 NAAL to acquire state-based data on adult literacy, and 15 states in addition to the District of Columbia and Puerto Rico used the 2016 Behavioral Risk Factor Surveillance System health literacy questions (Baer & Hsu, 2004; Centers for Disease Control and Prevention, 2017). These data describe people's skills aggregated at the state level. Measures of population literacy and health literacy skills are only one component, however, of how we can characterize community health literacy.

Community health assessments are another method from both public health and hospital-based community efforts that offer a framework for a “community health literacy” approach (Carlton & Singh, 2015). The Centers for Disease Control and Prevention Community Health Assessment and Health Improvement Planning website (2018) lists multiple planning tools. Although any group can conduct a community health assessment, local hospitals or health departments typically lead the assessment in a geographically defined area relevant to the organization(s). Health departments are now required to perform a formal community health needs assessment to apply for accreditation from the Public Health Accreditation Board (2013). Nonprofit hospitals are also required to perform periodic community assessments to maintain their nonprofit tax status, and the Affordable Care Act increased this requirement so that hospitals must conduct a community health needs assessment every 3 years, have an implementation strategy, measure the impact, and report annually to the Internal Revenue Service (Internal Revenue Service, 2018; Centers for Disease Control and Prevention, 2018; National Academy for State Health Policy, 2018). The National Academy of Medicine (2018) Collaborative Working Group on Community Health Needs Assessment Principles and Practices is developing guiding principles for hospitals, health departments, and community members to work together on community health assessments.

Although there is no single set of questions to conduct a community health assessment, in its most basic form the assessment involves gathering and analyzing data on community health indicators and issues to establish baselines, identify priorities, and inform future actions (Association for Community Health Improvement, 2017b; Centers for Disease Control and Prevention, 2015). This comprehensive approach can lead to community-wide change through strategy development, partnerships, research, and action, making this assessment a best practice (Barnett, 2012). An ideal process would also include a wide range of diverse perspectives with community members as full participants in all steps of assessment, planning, implementation, and reporting. An assessment includes multiple steps such as determining the purpose and scope of the assessment, identifying and collecting data on the health and needs of the selected community, understanding and interpreting the collected data, defining and validating priorities, and then documenting and communicating results (Catholic Health Association of the United States, 2013). The assessment can enhance organizational and community collaboration, identify community strengths and weaknesses, strengthen partnerships between and within state and local public health systems, and provide a benchmark for public health practice improvements (Centers for Disease Control and Prevention, 2015). Results are often used to justify resource allocation and inform strategic planning for widespread health issues, and ultimately create a community health improvement plan to systematically address the public health problems within a state or community (Centers for Disease Control and Prevention, 2015). From a health communication and health literacy perspective, a community health assessment should highlight the communication, information, and health education gaps, assets, and opportunities to incorporate health literacy strategies and tactics that improve individual, community, and population health literacy. The Maryland five-county rural health assessment is an example of channeling community members' public comments on health literacy into a recommendation for future investments and initiatives (University of Maryland, School of Public Health, 2017).

In an effort to explore and test a method for characterizing community health literacy, a team from the University of Maryland Horowitz Center for Health Literacy adapted the community health needs assessment framework to create a Community Health Literacy Assessment (CHLA) model for Maryland. The purpose was to build on existing community health assessments of Maryland counties and focus on the health literacy activities, assets, gaps, and opportunities in each county as well as for organizations serving broad regions or the state as a whole. Our process aligns with several steps in the Community Health Assessment Toolkit (Association for Community Health Improvement, 2017a). In this article, we focus on reporting our process and describe the steps to identify and engage stakeholders (Step 2), define the community (Step 3), collect and analyze data (Step 4), and document and communicate results (Step 6) (Association for Community Health Improvement, 2017a). The project's results will be discussed in a more detailed report in the future. The team did an environmental scan, document review, interviews, interview analysis and synthesis, and distribution and validation of findings. The specific objectives of this assessment were to do as follows:

- explore health literacy activities, assets, gaps, and opportunities at the county and state-level in Maryland;
- learn how county-level organizations rank themselves on their use of health literacy practices;
- create a network of public health professionals dedicated to sharing and improving health literacy practices and examples across Maryland.

In this article we offer a framework that organizations at the state level (such as a state health improvement process or state departments of health), county level (county departments of health, local health improvement coalitions), and community level (hospitals, nonprofits) can replicate to collect primary information and examples of health literacy activities, assets, gaps, and opportunities. The information can serve three purposes: (1) show organizations where they have focused their attention and where they can improve their health literacy practices; (2) influence future research and interventions; and (3) inform strategic planning at the organizational, county, and state-levels.

## Brief Description of Activity

A team from the Horowitz Center for Health Literacy, which included the Center's Director and six students from the University of Maryland (two doctoral candidates, one Master of Publich Health candidate, two undergraduates, and a communications undergraduate intern), worked on this project from January 2018 to April 2018.

The team created and conducted a CHLA framework that defined “community” in two ways. One was geographic by county and the other was by populations served within a county. The CHLA included an environmental scan, document review, interviews, information analysis and synthesis, and distribution and validation of findings. An environmental scan was completed to obtain background data on each county, such as county health indicators and population demographics. Document review included reading and extracting pertinent information from various reports and documents for each county. The team created and pilot tested a semi-structured questionnaire to interview participants who work at local coalitions, health departments, federally qualified health centers, faith-based organizations, state government agencies, nonprofits, and hospitals. Interview questions focused on the populations served, priority health concerns, health literacy activities, assets, and health literacy needs. Participants were also asked to rate their use of health literacy practices.

Over a 2-month period, 56 semi-structured interviews were conducted with 49 different organizations, 11 of which work statewide. After completing interviews and interpreting the information from each interview, a forum was held to share results with participants and public health professionals across the state.

## Implementation

**Figure 1.**
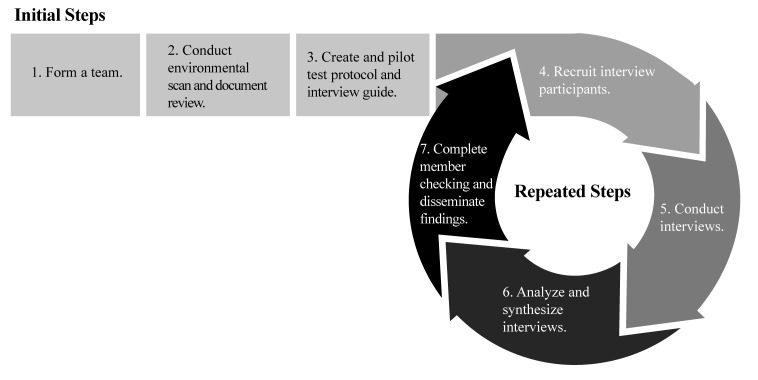
This figure shows the initial steps of the Community Health Literacy Assessment framework that coalitions can complete to collect information on health literacy for a community, county, region, and state.

This project was implemented from January 2018 to April 2018. The planning period, environmental scan, and document review took place from January to February, with interviews starting in mid-February and lasting until early April. Review and interpretation of the interviews started in March and lasted until mid-April. A culminating forum occurred in late April to share results and perform member checking. Member checking is a best practice in which results or interpretations of collected information are reviewed with participants and community members to assess accuracy (Charmaz, 2014). A flowchart demonstrating the steps of the CHLA framework can be found in **Figure 1**. The framework is for organizations that aim to conduct a health literacy assessment within a community, county, region, or state.

### Environmental Scan and Document Review

The team conducted an environmental scan to gather publicly available background data on the 24 counties in Maryland, including the city of Baltimore. A template for each county was used to systematically record relevant information discovered through the Internet and database searches. Internet and database searches were completed to collect information on county health indicators and population demographics, find relevant county reports and documents, and identify important health organizations, county policies, available community resources and services, public health professionals, and county opinion leaders. County indicators included county resources such as local libraries, health services, and community health workers. Population demographics, economic factors (county employment, education, median income), physical environment (transportation, environmental setting, population size), and health indicators were also researched. Terms used for Internet searches included population demographics, community health needs assessment, hospital utilization data, and community health services, among others. The name of the county was placed in front of each term being researched.

The team started by examining existing community health needs assessments when we were able to locate them. We also found data from county census databases and by state and county planning and assessment teams who have implemented State Health Improvement Processes as a framework to evaluate and enhance community health (Maryland Department of Health, 2019). The team examined county and community health reports to determine how health literacy is incorporated in each county. This process allowed the team to better understand each county, such as their health priorities and any existing health literacy activities, before starting interviews.

### Interview Protocol and Semi-Structured Interview Guide

An interview protocol and semi-structured interview guide were created to improve fidelity as well as guide conversation with interview participants. The protocol included an email template to schedule interviews, a list of health literacy “buzzwords,” and a short template to use while talking to local coalition staff about accessing their membership list for potential interviews. The interview guide included information on what to say before the interview, including an introduction of the interview team and the Horowitz Center, as well as an overview of the project. It also included a verbal consent request to transcribe notes taken during the meeting, the interview questions, and how to conclude the interview. Written consent was not required from participants because the University of Maryland's Institutional Review Board provided exempt status for this project (IRBNet ID 1192606-1). The project team completed Collaborative Institutional Training Initiative classes for human subjects research and was trained on how to use the interview protocol, interview guide, and interviewing techniques such as how to pick up on cues to ask appropriate probing questions.

### Interview Questions

Although there is no standard set of community health needs assessment questions, typical questions probe the health indicators, activities, resources, opinions, perceptions, and related factors that affect a community's health (Association for Community Health Improvement, 2017a). The team used a similar approach and drafted and pilot tested interview questions with two staff members from a local health improvement coalition managed by a county health department. Questions were assessed for understandability, flow, and clarity. Feedback was incorporated to improve and finalize the questions, which included items on populations served, priority health concerns, health literacy activities, assets, and health literacy needs. Questions were meant to capture participants' knowledge of health literacy activities, assets, gaps, and opportunities as well as organizational behaviors related to health literacy. Example activities can be seen in **Table A**. Assets were defined as factors that do or could support health literacy work or progress; gaps were defined as missing factors inhibiting work or progress; and opportunities were defined as factors that could advance work or progress if acted on. The final 13 questions, as well as probes and follow-ups, can be seen in **Table 1**.

### Interview Participant Recruitment

Participants for county interviews were recruited through local health improvement coalitions and existing Horowitz Center contacts. The Maryland Department of Health (2016) requires each county to have a local health improvement coalition (LHIC), which is a group of jurisdictional-level stakeholders and leaders from community-based organizations, government agencies, and health care providers who collaborate to determine public health priorities within their community and address local health priorities through creating programs and policies with support from the county and state. By targeting each LHIC in Maryland, the team was able to identify engaged public health professionals in each county who create and enact strategies to address critical health needs, which may include health literacy. Potential participants were contacted by email using a template that explained the project and requested an in-person or over-the-phone interview. “Snowball” sampling methods were also used, as participants were asked to identify other potential participants in their county at the end of each interview. Specifically, participants were asked if they could refer us to someone who could further discuss the health literacy activities in their county. For state-level interviews, potential participants were based on past contact with the Horowitz Center or through contacts of the Center Director.

### Interview Procedures

After completing the environmental scan and document review, the team conducted semi-structured interviews with representatives from LHICs and other state and county organizations. Every LHIC in the state was contacted, but not all of them responded to our interview request. The team interviewed at least one representative from a key organization per county. We completed interviews in person or over the phone if participants were located more than 60 minutes away from College Park, MD, or if the participant preferred a phone interview. Interviews ranged from 30 minutes to 1 hour. Detailed notes, similar to a transcript, were taken during each interview. After the interview, the team member(s) who conducted the interview reviewed the notes and wrote a brief report summarizing the answers to the interview questions. Health literacy assets, gaps, and opportunities as well as the various health literacy-related services or resources available to county and state residents were detailed in the report.

### Interview Analysis and Synthesis

Interview notes were compared and reviewed to identify common themes such as county assets regarding health literacy, gaps in resources in the county, and opportunities to improve organizational health literacy. Assets, gaps, and opportunities were distinguished by each participant's interview answers. For example, questions 5 and 9 (“What does your organization already do to address health literacy in your county?” and “What are the assets in your county to address or support health literacy?”) were related to assets. Question 10 (“What kind of health literacy resources or services do you believe are needed in the state?”) often resulted in the participant naming gaps and opportunities to fill those gaps. We also included a follow-up to question 8 (“What do you hope to accomplish by addressing health literacy in your county?” and “What prevents you from doing so?”) to assess gaps.

### Distribution and Validation of Findings

The Horowitz Center for Health Literacy hosted a forum called the “Health Literacy Huddle: A Dialogue on Health Literacy in Maryland” in April 2018 to confirm and disseminate the findings gathered during the needs assessment process. More than 70 public health professionals were invited to attend the forum in person or through online streaming. Results were presented through four poster displays and a 1-hour presentation with a discussion component. In addition to disseminating the results, the project team was able to perform member checking with the audience, which is when results are shared with participants to assess their agreement with research findings and interpretations of results (Charmaz, 2014). There are many ways in which member checking can be completed, such as one-on-one visits or phone calls, focus groups, or through presentation of results at a forum (Creswell & Miller, 2000). After the event, project results were posted on the Center's website, which provided another opportunity for member checking. After the presentation, a lunch was served and forum participants were able to network. An evaluation was also shared with participants by email after the forum to determine what information was most valuable and what participants would like to learn at a future event, and to evaluate their interest in future health literacy conferences.

## Results

This article reports results related to our process, and a future article will report detailed results of the information we collected. The team was successful in gathering an initial round of information about health literacy activities, assets, gaps, and opportunities in each county in the state of Maryland. There were 49 organizations interviewed for a total of 56 interviews in a 2-month period. These 56 interviews cover all 24 counties in the state of Maryland. A minimum of two interviews were completed in 18 of the 24 counties in Maryland. In the remaining six counties, the team spoke with a leader from each county local health improvement coalition, which represents a collection of interests from that county. Forty-five interviews were conducted with 38 county-level organizations. Types of organizations included health coalitions, health departments, community health centers, hospitals, cooperative extension, nonprofit organizations, literacy councils and public libraries, and faith-based organizations. There were 11 state-level organizations interviewed, which included governmental (including the state department of health) and nonprofit organizations. Of the 56 interviews, 18 were completed in person, 36 on the telephone, and 2 through email. When comparing each interview method, we gathered the most information through in-person and telephone interviews.

Participants were asked to rate their organization's use of health literacy techniques and best practices on a scale from 1 to 5, with 1 being *very inadequate* and 5 being *highly adequate*. This question was answered in 40 of the 45 county-level interviews and 5 of the 11 state-level interviews. The self-rankings of statewide organizations ranged from 3 to 4, with 3.5 as the average. The average county organization self-ranking ranged from 2 to 4.67. Individual county-level interview participants ranked their organization's use of health literacy techniques and best practices as followed: 1 (*n* = 0); 2 (*n* = 13); 3 (*n* = 13); 4 (*n* = 11); 5 (*n* = 3), and no ranking provided (*n* = 5).

Participants were asked what health literacy meant to their organization. Descriptions were first coded on whether they were focused on the individual patient level, the systems/organizational level, the community level, the policy level, or a mixture of the four. Of the 45 county interviews, 40 descriptions discussed health literacy at the individual or patient level, nine at the community level, five at the systems/organizational level, and zero at the policy level; two participants did not provide a description. Several county participants (*n* = 10) described health literacy in multiple ways, with five as individual and community level, three as individual and systems level, one as community and systems level, and one as individual, community, and systems level. State organizations (*n* = 11) described health literacy at the individual level (*n* = 10); systems level (*n* = 4); community level (*n* = 2); and policy level (*n* = 1). Five of the 11 state-level participants described health literacy in multiple ways: systems/individual (*n* = 2); systems/policy (*n* = 1); individual/community (*n* = 1); and individual/community/systems (*n* = 1). The statements were also coded for how participants described health literacy; the most common code was “understanding,” followed by “patient communication” (**Table 2**). The team did not evaluate participants' health literacy descriptions for alignment with standard health literacy definitions.

Forty public health professionals attended the forum in-person or virtually. Ten interview participants attended the forum, and many brought other members of their organization with them. Member checking with the attendees found that interview participant descriptions and the overall picture of health literacy across the state were accurately portrayed.

The forum was successful in stimulating discussion about the results and the future of health literacy in Maryland. Fourteen attendees completed the evaluation, which showed that the most valuable part of the forum was hearing the results of the project, followed by the ability to network with other organizations interested in health literacy. Future topics participants would like to learn included follow-up on this project, best practices in health literacy, funding opportunities for health literacy activities, and health literacy training opportunities, such as how to evaluate health materials or design health messages for specific populations.

The environmental scan of existing reports and health data did not provide much information regarding health literacy activities, assets, gaps, or opportunities. This could be due to limited health literacy data and information publicly available at the community and organizational level, demonstrating a need for CHLAs. However, the environmental scan was helpful in educating the team on each county and preparing us for interviews, so our team still recommends this activity before starting the interview process.

The project team was able to successfully gather preliminary data on the state of health literacy in Maryland, confirming that this health literacy assessment structure works. The team established an initial view of health literacy activities, assets, gaps, and opportunities at the county and state level, learned how county-level organizations rank themselves on their use of health literacy, and created a network of public health professionals dedicated to sharing and improving health literacy across the state. The findings also identified support for relaunching a statewide collaboration that had stalled because of lack of funding and staffing. Additionally, a Health Literacy Maryland Steering Committee was recently created, which includes a participant from this project. The committee seeks to promote health literacy in Maryland through educating a coalition of community leaders and mobilizing them into action within their jurisdictions. Committee members come from diverse geographical areas within Maryland and work in government positions, university libraries, and community health centers. The committee meets quarterly to discuss how Health Literacy Maryland can support organizations in integrating health literacy concepts and practices. Furthermore, the Horowitz Center hosts webinars and conferences to offer professional development opportunities to organizations across the state to build a common understanding of health literacy and activities. Many organizations who participated in this project are now using these professional development resources, which is strengthening the network of health professionals advancing health literacy in Maryland.

## Lessons Learned and Recommendations

Capturing lessons learned from a project is a valuable method to describe what went well, what could have been improved upon with recommendations for refined replication, and how other groups can benefit from implementing the same or similar projects. This project demonstrates that a small team using a structured CHLA framework can collect a significant amount of information at the county and state level in a short period of time. This project used an exploratory approach to health literacy, meaning that participants described what health literacy meant to them or their organization. However, if the participant was unclear on “health literacy” and asked for clarification, we shared the Horowitz Center's working definition about making it easy for everyone to find and use information and services for health and well-being. This exploratory approach to health literacy is recommended for conducting interviews to understand how different types of organizations describe health literacy and what activities or procedures participants believe are related to health literacy. We found uncertainty about the boundaries of health literacy; therefore, we recommend not defining the term so participant responses are not limited or influenced. In addition to the multiple descriptions of health literacy, we found a range of self-report scores for rating organizational use of health literacy techniques and best practices. We recommend future interviews include a checklist of activities to evaluate health literacy as there is currently no definition or description of what constitutes a health literacy activity. It is important to remember the limits of self-report information; therefore, having clear objective criteria for measuring health literacy activities could assist organizations in ranking their use of health literacy techniques and best practices. A health literacy activity checklist based on interview responses is included in **Table A**.

Interviews were conducted in person as well as over the phone so that remote parts of the state could be reached. We also allowed email responses when it was too difficult to schedule a phone call. We found that phone interviews were less conversational and closely followed the interview guide, whereas in-person interviews were more conversational. Email responses only allowed for the main interview questions to be answered, without the opportunity to prompt participants or ask follow-up questions. We recommend that teams be flexible in their approach to collecting information, especially if they are examining a large geographic area. Having multiple options, such as phone interviews or email replies, ensures that information collection is as inclusive as possible. Furthermore, if an email response or over-the-phone interview is not as fruitful as hoped, follow-up questions sent by email or on a phone call can be asked to gather more information.

Throughout the interview process, the team realized that the interview questions were better suited for county-level concerns than regional or state operations. Teams that use the CHLA in diverse settings may consider having different sets of questions depending on their populations of interest. Additionally, questions can be updated or added if a participant discusses something relevant that is not covered in the interview guide. Previous participants can also be contacted to ask new questions, or they can be discussed during the member checking process.

Member checking is a validation process included in this framework and is recommended to assess participant agreement with the results of a project. Forum participants were able to contribute their thoughts on the accuracy of the results in person as well as online via WebEx streaming or on the project's website. Multiple options were presented to expand dissemination of the results as participants were located all over the state.

Teams can use this systematic framework (**Figure 1**) to collect preliminary data on health literacy as well as continue the process to attain a health literacy baseline at different operational levels. To collect preliminary data on health literacy, we recommend completing the framework cycle at least one time. To achieve a baseline of health literacy, we recommend completing steps 1 to 3, then repeating steps 4 to 7 until saturation is reached. Saturation is accomplished when no new information is learned in subsequent interviews (Charmaz, 2014).

The results of this project are being used to inform strategic planning for the Horowitz Center, Health Literacy Maryland, and three county health departments. The Center is working with the health departments to create and pilot test health literacy logic models and brief action reports. The expectation is the logic models will be implemented in each health department to integrate health literacy in current community programs and activities.

The CHLA framework gives state, county, and community organizations the opportunity to examine how health literacy is understood and being implemented locally. To our knowledge, this is the first systematic framework to examine how multiple organizations use health literacy techniques across communities. Our results suggest that other communities may find this a similarly useful process. We recommend that county and state organizations use the CHLA framework to help achieve the National Action Plan's goal of a health literate society.

## Figures and Tables

**Table 1:** Semi-Structured Interview Questions

**Number**	**Interview Questions**	**Prompts and Follow-Up Questions**
1	How do you describe the populations you serve?	-
2	What health issues does your organization consider to be a priority in your county?	-
3	I understand that your organization's mission is X.^a^ How does that relate to health information and services for consumers and patients?	Probe: Could you give an example of educational, informative, knowledge, or skill-building activities that you implement, such as health information seeking skills or how to communicate with health care professionals? Follow-up: What is the process used to develop those activities, materials, or skills?
4	What does health literacy mean to your organization?	Probe: If not clear for individual, insert definition from the center: “Make it easy for everyone to find and use information and services for health and well-being”
5	What does your organization already do to address health literacy in your county?	Probe: How are you developing activities/materials/skills for the populations (race/ethnicity/age/literacy skills) you currently serve?
6	Using a scale from 1 to 5 with 1 being *very inadequate* and 5 being *highly adequate*, how would you rate your organization's use of health literacy techniques and best practices?	See **Table A** for health literacy activities list derived from participant responses. This list can be used by organizations to rate their use of health literacy techniques and best practices
7	What are the approaches or methods you use to identify health literacy concerns in your county?	Probe: What do you think causes these issues or problems? Please describe who it affects and the consequences Follow-up: What does your county do to avoid health literacy issues?
8	What do you hope to accomplish by addressing health literacy in your county?	Follow-up: What prevents you from doing so?
9	What are the assets in your county to address or support health literacy?	Probe: What programs, resources, or interventions are being implemented to address health literacy?
10	What kind of health literacy resources or services do you believe are needed in the state?	-
11	If we want to make Maryland a health-literate state, how can we start in your county?	-
12	How does your organization fund the health literacy services you provide?	Probe: What about long-term/short-term grants? Has your organization applied for federal or state funding? What sources do you use to find funding?
13	Who else in the county or the state do you suggest we talk to about health literacy?	-

Note.

a The mission of each organization interviewed was mentioned for this question.

**Table 2:** Participant Descriptions of Health Literacy (*N* = 56)

**Code**	**County Response (*n* = 45)**	**State Response (*n* = 11)**	**Example**
Understanding	32	5	“Health literacy is about people's ability to understand the medical information that they are given, process it appropriately, and then act on it”
Patient communication	16	4	“Means communication with our patients in the way that they want”
Access	14	6	“Residents know how to access care and how to access the appropriate level of care for whatever issues they are having”
Actionable activity	12	4	“Offer hands-on programs and visual programs and hand them information”
Plain language	12	0	“Communicating in a way that our clients understand, and being mindful of that, and helping them understand”
Empowerment or self-advocacy	10	2	“Awareness and being a self-advocate are very important”
Reading level	5	0	“Making the materials understandable, readable, visually readable and low reading level, using a variety of pictures and words”
Tailored information	5	1	“That might entail handwriting instructions, managing education regimens, and answering questions”
Offer materials in multiple languages	3	0	“We try and develop communication materials so that multiple audiences can understand, such as reading levels and having multiple languages”
Social determinants	3	0	“Understanding the social determinants of health for individuals”
Jargon only	2	1	“Can they get information, and can they understand it? Or is it above their level of understanding, full of jargon using a lot of medical terms?”
No description provided	2	0	Two participants did not answer this question

**Table A:** Heath Literacy Activity

**Activity**	**Example**
Educational videos	Share videos that provide nutrition and exercise demonstrations or teach residents about managing different health conditions using language that is easy to understand
Educational materials	Provide health education materials, such as self-management guides, newsletters, brochures, personalized instructions, booklets, and social media that can be evaluated or created using the Centers for Disease Control and Prevention (2019) Clear Communication Index
In-person community outreach	Offer targeted or tailored health education through disease prevention/management classes, home visits, mobile care units, forums, and one-on-one training
Raise provider awareness of health literacy	Share news about health literacy trainings, attend continuing education seminars, and advocate that providers and staff use clear communication
Provider health literacy training	Mandate training hours, incorporate clear communication techniques, and implement train-the-trainer strategies
Interpreter services	Provide interpretation services for languages commonly spoken in the area and/or American Sign Language
Multiple languages	Provide websites, health classes, and offer health education materials in multiple languages
Community input and engagement	Host community focus groups to evaluate materials, use community members to create health messages, have community members involved in steering committees and/or review boards
